# Bridging Science Across Species: A Biomechanics Outreach Event at the Zoo

**DOI:** 10.1093/iob/obag022

**Published:** 2026-05-19

**Authors:** Cassie Shriver, Audra Davidson, Henry C Astley, Saad Bhamla, Kai L Yung, Danielle S Adams, V David Munteanu, Richard W Blob, Charles Hammer, Skylar Taylor, Young-Hui Chang, David L Hu, Michelle Kolar, Staci Wiech, Joseph R Mendelson III, Andrew K Schulz

**Affiliations:** Department of Biological Sciences, Georgia Institute of Technology, Atlanta, GA 30332, USA; Department of Biological Sciences, Georgia Institute of Technology, Atlanta, GA 30332, USA; Department of Biology, University of Akron, Akron, OH 44325, USA; Department of Chemical and Biomolecular Engineering, Georgia Institute of Technology, Atlanta, GA, 30332, USA; Department of Ecology and Evolutionary Biology, University of California, Irvine, CA 92697, USA; Field Museum of Natural History, Chicago, USA; Department of Biological Sciences, Clemson University, Clemson, SC 29634, USA; Department of Biological Sciences, Clemson University, Clemson, SC 29634, USA; Department of Medicine, Emory University, Atlanta, GA 30322, USA; Department of Ecology and Evolutionary Biology, University of California, Irvine, CA 92697, USA; Department of Biological Sciences, Georgia Institute of Technology, Atlanta, GA 30332, USA; Department of Biological Sciences, Georgia Institute of Technology, Atlanta, GA 30332, USA; Department of Mechanical Engineering, Georgia Institute of Technology, Atlanta, Ga 30332, USA; Indianapolis Zoo, Indianapolis, IN 46222, USA; Zoo Atlanta, Atlanta, GA 30315, USA; Department of Biological Sciences, Georgia Institute of Technology, Atlanta, GA 30332, USA; Zoo Atlanta, Atlanta, GA 30315, USA; Department of Mechanical Engineering, Georgia Institute of Technology, Atlanta, Ga 30332, USA; Haptic Intelligence Department, Max Planck Institute for Intelligent Systems, Stuttgart 70569, Germany

## Abstract

Outreach events are an excellent way to expose science disciplines to the public beyond the ivory tower of academics. In this paper, we describe how a zoo-academia collaborative team designed and hosted an interdisciplinary outreach event exposing thousands to the wonders of bio-inspired design and biomechanics. Over the past 4 years, we have run such a collaborative outreach event which has attracted 26,750 people, allowing children and adults to be exposed to diverse research fields through our annual Zoo Biomechanics Day. In partnering with labs, companies, and student organizations, we have hosted 17 unique booths highlighting human, plant, and animal biomechanics and bio-inspiration to showcase engaging demonstrations of science, technology, engineering, arts, and medicine principles. This event is low-cost ($\< \$300$ annually) while increasing attendance at the zoo by over 65% compared to similar non-event days. Furthermore, participating researchers get connected to the zoo, allowing discussions of new collaborative research between the zoo and universities. This paper will serve as a framework for others to host events which highlight the zoo-university collaborations that enhance the dual objectives of outreach and research at modern zoos.

## Spanish

Los eventos de divulgación son una excelente forma de exponer disciplinas científicas al público, más allá de la torre de marfil de los académicos. En este artículo, aprovechamos un equipo colaborativo entre un zoológico y la academia para organizar un evento de divulgación interdisciplinario exponiendo a miles de personas, las maravillas del diseño bio-inspirado y en la biomecánica. Durante los últimos cuatro años, hemos organizado un evento colaborativo de divulgación que ha atraído a mas de 26.750 personas, permitiendo que niños y adultos estén expuestos a diversos campos de investigación a través de nuestro Día anual de la Biomecánica del Zoológico. En colaboración con laboratorios, empresas y organizaciones estudiantiles, hemos montado 17 puestos únicos dedicados a la biomecánica humana, vegetal y animal, así como a la bioinspiración, con el fin de ofrecer demostraciones atractivas de los principios de la ciencia, la tecnología, la ingeniería, las artes y la medicina (STEAM). Este evento es de bajo costo (<US$300 anuales) y aumenta la asistencia al zoológico en más del 65% comparado a días similares sin eventos. Ademas, los investigadores participantes crean una conexión con el zoológico, permitiendo las discusiones de nuevas investigaciones colaborativas entre el zoológico y las universidades. Este artículo servirá como un marco para otros organicen eventos que destaquen las colaboraciones entre las universidades y zoológicos que potencian los objetivos duales de la divulgación e investigación en los zoológicos modernos.

## German

Outreach-Veranstaltungen sind eine hervorragende Möglichkeit, wissenschaftliche Disziplinen über den Elfenbeinturm der Wissenschaft hinaus der Öffentlichkeit zugänglich zu machen. In dieser Publikation nutzen wir ein gemeinschaftliches Team aus Zoos und Wissenschaft, um eine interdisziplinäre Outreach-Veranstaltung zu organisieren, die Tausenden von Menschen die Wunder des bioinspirierten Designs und der Biomechanik näherbringt. Vergleichbare Veranstaltungen innerhalb der letzten vier Jahre zogen über 26.750 Menschen an und ermöglichten Kindern und Erwachsenen einen Einblick in verschiedene

Forschungsbereiche durch unseren jährlichen Zoo-Biomechanik-Tag. In Zusammenarbeit mit Labors, Unternehmen und studentischen Organisationen haben wir 17 einzigartige Stände organisiert, die sich mit der Biomechanik von Menschen, Pflanzen und Tieren sowie mit Bio-Inspiration befassen, um die Prinzipien von Wissenschaft, Technologie, Ingenieurwesen, Kunst und Medizin (STEAM) auf anschauliche Weise zu veranschaulichen. Diese Veranstaltung ist kostengünstig (weniger als 300 Dollar pro Jahr) und steigerte die Besucherzahlen im Zoo um über 65% im Vergleich zu ähnlichen Tagen ohne Veranstaltung. Darüber hinaus knüpfen die teilnehmenden Forscher Kontakte zum Zoo, was Diskussionen über neue Forschungskooperationen zwischen dem Zoo und den Universitäten ermöglicht. Das Ziel dieser Publikation ist, als Rahmen für interessierte Parteien zu dienen, um Veranstaltungen zu organisieren, die die Zusammenarbeit zwischen Zoos und Universitäten stärken, sowie die Öffentlichkeitsarbeit und Forschung in modernen Zoos fördern.

## Introduction

Modern science is integrative, leveraging several different fields to answer interdisciplinary or even transdisciplinary questions (Van Noorden 2015; Schulz et al. 2024). However, as science blurs the lines between these disciplines, the barrier-to-entry for less traditional fields increases significantly (Pischke et al. 2017). Traditional disciplines, such as engineering or biology, have several examples of interdisciplinary collaborations where teams from both fields work together to understand and solve novel questions (Calvert 2013; Hashemi Farzaneh 2020; Schulz et al. 2023). Bio-inspired design takes inspiration from the structure-function relationship in nature to inform designs for human technological advancement (Shu et al. 2011). Biomechanics uses engineering techniques such as material testing, modeling, and manufacturing to augment, repair, or understand how the structure–function relationship changes with age or pathologies (Lu and Chang 2012; Robertson et al. 2013) as well as how such structure–function complexes evolve (Astley et al. 2020). While biology and engineering, in their modern-day conceptions, emerged as formalized fields and professions in the 18th and 19th centuries, it wasn’t until the late 20th and 21st centuries that interdisciplinary mergers of these two appeared (Porter and Rafols 2009). Biomechanics and bio-inspired design are often introduced to students in the late undergraduate curriculum (Riskowski 2015; Nagel and Pidaparti 2016). Because students have already nearly completed their undergraduate degrees prior to these courses, this late appearance in a student’s education causes a significant barrier-to-entry for them to understand the possibility that such research can be accomplished, or that it represents a potential path for graduate school or a career.

One way to tear the walls down on fields with a barrier is through early education, outreach, and engagement (Wang 2013). Educators have worked to introduce concepts such as cross-disciplinarity early in college with project-based curricula for active learning (Clyne and Billiar 2016). Still, these often impact students too late to have an effect on their post-graduate plans. Significant

successes have occurred with large-scale outreach events that have helped promote interdisciplinarity and engage a wide and diverse audience (Beery et al. 2022). Outreach events such as university open-house days, science festivals, or discipline-specific events such as National Biomechanics Day have worked to bring interdisciplinary science to the broader community at large (Aoki et al. 2026).

National Biomechanics Day (NBD) was founded in 2018 to bring biomechanics to all ages through outreach events hosted worldwide and throughout the year (DeVita 2018). These events feature a wide range of outreach opportunities, and each event hosts tens to hundreds of individuals, totaling several thousand people learning about biomechanics throughout the year (DeVita 2023). NBD events range from showcasing the intricate biomechanics of dance (Zaferiou 2023) to highlighting diversity and equity disparities in biomechanics (Kirk et al. 2023). Outreach helps STEM students see the possibility of pursuing new and innovative scientific disciplines, and organizations such as Girls Who Code and Women in Robotics also work to plug the leaky pipeline of scientific disciplines, exposing a field to those less privileged or lacking a support network or role models so that they can experience robotics (Sullivan et al. 2015).

While NBD is internationally successful in creating large biomechanics outreach events, we shifted the scope in our current model to highlight academic research made possible with collaborations with non-academic institutions, such as zoos, aquariums, and museums. A mission of zoos for the past century has been the exposure of attendees to the wonders of conservation and exposing the public to animals they would not be able to see in the wild, as well as contribute to general biological knowledge (Mendelson et al. 2019). A more recent mission of modern zoos is to work on research with the species housed in zoological settings to help inform and conserve species in the wild. Zoo-university collaborations allow this connection between research, conservation, and innovation (Schulz et al. 2022). In this paper, we describe how people from academia and a zoo collaborated to create a large-scale outreach event for the public to learn about form–function relationships in animals, to expose teachers and students to the methods, techniques, and to highlight a range of zoo-interfaced research fields, including biomechanics, bio-inspired design, and more. The largest zoos can host tens of thousands of people in a day during peak hours. By collaborating with one of these organizations, outreach events can have their impact increased 10-fold compared to hosting at a local university.

## Methods

### Establishing academic-outreach partnerships for the event

Georgia Tech and Zoo Atlanta have had a strong academic partnership for over 30 years spanning dozens of papers, and ongoing research, outreach, service, and educational events. The first step of executing this event was a positive and collaborative partnership, which we have previously discussed in guidelines for creating a zoo–academia partnership (Schulz et al. 2022). Leveraging this partnership in 2019, we began the process of planning and executing a zoo-academic outreach event in the Atlanta area to be hosted in the spring of 2020; however, the event was postponed to start in the Spring of 2023 because of the COVID-19 pandemic.

### Planning zoo-academic biomechanics events

To increase awareness, we partnered with Atlanta Science Festival, an annual, 2-week-long festival that promotes and hosts events centered on providing scientific outreach for K–12 students. For our event, we reached out to research laboratories working on one or more of our four topical areas: morphology, bio-inspired robotics, biomechanics methods, and comparative biomechanics. In the first year, we reached out to the local community of scientists conducting research within the aforementioned topical areas. In the following years, we contacted labs that had previously participated, and we reached out more broadly geographically to welcome additional labs expressing interest. Later years additionally featured participation from student organizations highlighting all parts of the science, technology, engineering, arts, and medicine (STEAM) pipeline.

**Fig. 1:**
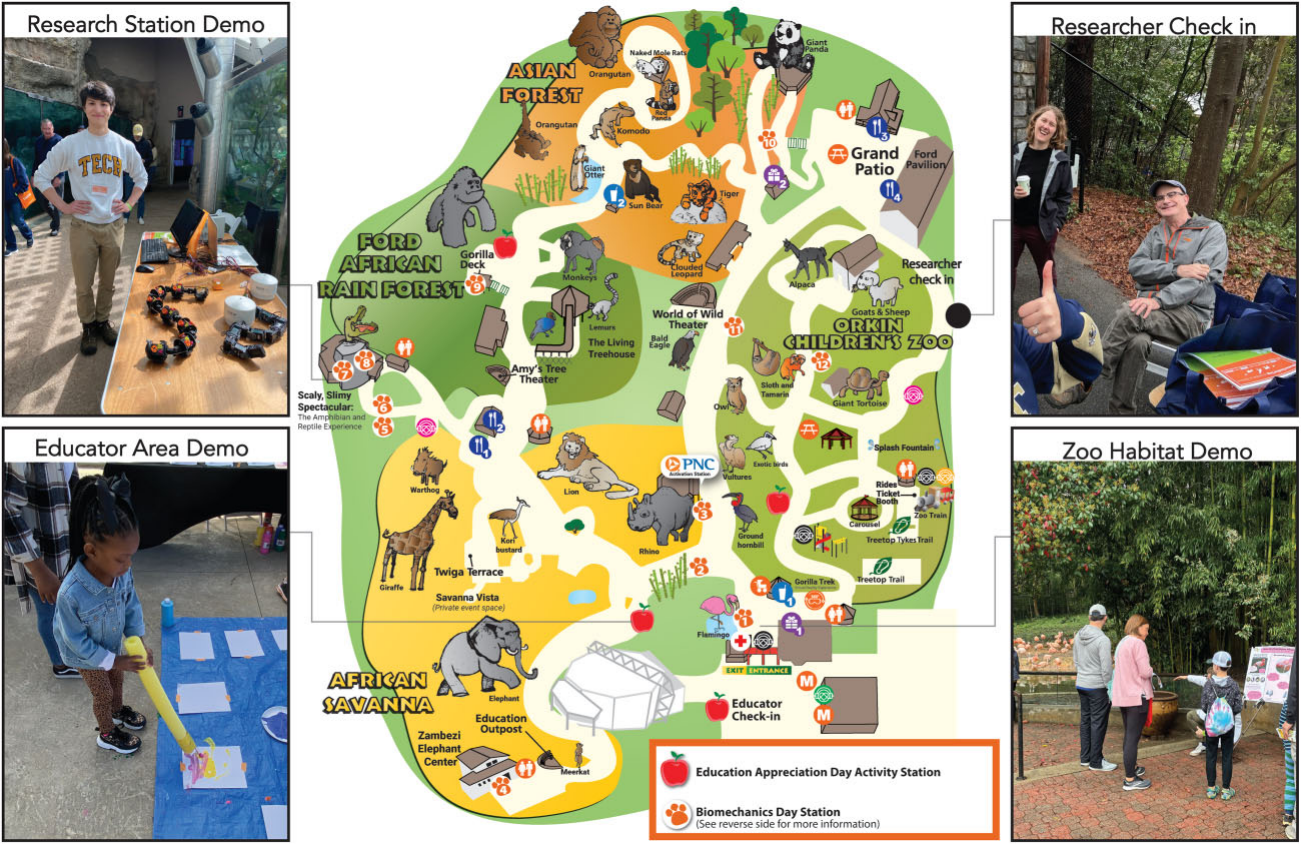
Animals in Motion: Biomechanics Day at the Zoo layout. Overall layout of the zoo during the event highlighting specific contributions making it possible including the zoo habitats with educator stations, marked with apples, as well as researcher stations, marked with paw prints. Researcher station demos describe stations, like this bio-inspired snake robot, which is not directly paired with a species allowing researchers to give flexible demonstrations around parts of the zoo. Educator-area demos were run by Zoo Atlanta educators and featured activities readily adaptable to classrooms, such as trying to paint with an elephant “trunk.” The researcher check-in booth was for researchers who were getting to the zoo to get all of the items necessary for their specific tables. Finally, zoo-habitat demos were specific to a particular habitat with this example happening right in front of the flamingo portion of the zoo.

In order to participate, interested research labs needed to create a booth with an intended message for their audience and generate a corresponding description. Because zoo guests ranged in ages and education levels, we also worked with education experts at Zoo Atlanta to review descriptions and ensure they were suitable for a broad audience. Finally, the event was partnered with Educator Appreciation Day, an annual event at the zoo designed to draw in local educators and allowing teachers to learn about educational resources the zoo offers. To this end, Zoo Atlanta also created additional stations throughout the zoo focusing on education. These two types of stations, staffed by university researchers and zoo educators, combined to form our event named “Biomechanics Day: Animals in Motion” (Fig. 1). To finalize the design and the placement of booths from participating labs, we worked with researchers to understand their needs. We first determined specific station needs, such as the amount of space and number of tables requested and whether they needed access to electricity or a covered area. We then set out to place stations in locations that would both meet their needs and be near relevant species, if possible. This included human biomechanics labs being near the Western Lowland Gorilla exhibit, or a snake robot being outside a snake habitat. The zoo was crucial in determining which locations were most suitable and also coordinated setting up tables and chairs for the stations the evening before or morning of the event.

The event was promoted and advertised through social media, blog posts by the scientists that were participating, the universities involved, and the Atlanta Science Festival website. Zoo Atlanta also created a page on their website for participants to learn more information prior to the event, for both biomechanics research and Educator Appreciation Day.

### Running Zoo Biomechanics Day

**Fig. 2:**
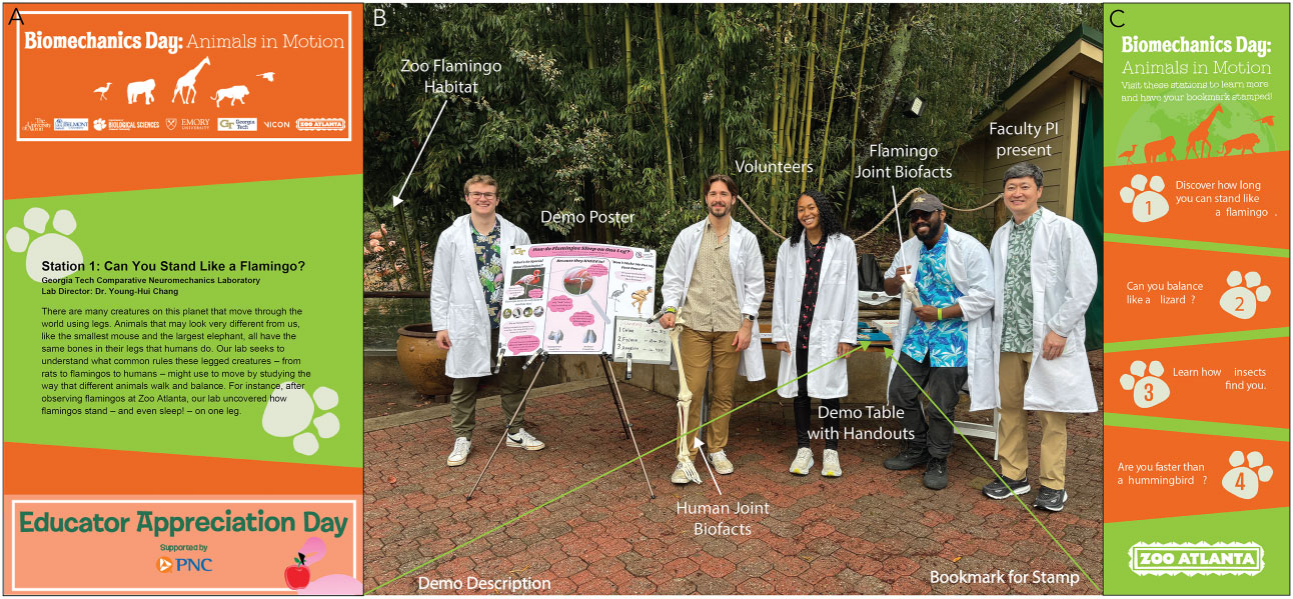
Example layout of a table with booth information. This figure showcases the table items provided for every scientific volunteer booth. (A) The document at each station showcasing the description of the event and what to learn. (B) Example of a booth for how to stand like a flamingo showing that the booth is in front of the habitat with bio-facts, volunteers, and the lab principal investigator (PI), (C) Bookmarks given to participants that could get stamps at different booth for a final reward if children visited each booth around the zoo.

For each event’s year, researchers checked-in and set up an hour before the zoo opened to the public. Overall, the event ran each year for four total hours. Each station was provided stacks of handouts, including specialty maps to show where the booths were throughout the zoo, flyers with more detailed descriptions of the research and animals represented in the booths, and bookmarks (Fig. 2). The bookmarks listed the various station numbers with a brief topical blurb, and each station was provided stamps for guests to mark off the respective number after visiting it. Every station was also customized to the researchers/educators specifications. Many of the stations had a poster highlighting their work or included bio-facts, such as 3D printed skulls, to show morphology. There were also demonstrations where participants were able to attend and interact with high-speed cameras. At the conclusion of the biomechanics event, we had researchers bring all materials out of the zoo at the same location they arrived and collected the stamps, station signs, and leftover papers for recycling. Over the course of the 4 years, we have had 17 unique booth setups around the zoo hosting various researchers to highlight flora and fauna (including humans).

### Evaluating participation and attendance numbers

After the conclusion of each annual event, we are able to assess the number of participants. Numbers of participation are compared to previous years and other weekend attendance numbers for the given weather, as weather is highly correlated with zoo attendance (Perkins and Debbage 2016). During the 2023, 2025, and 2026 years, the weather was sunny and during the 2024 year, the entire day was rainy without sun. For the number of participants, we were able to separate attendance numbers based on zoo ticket sales. Children under the age of 3 are not included in this number.

## Results

### Designing biomechanics stations

Once research labs confirmed interest and availability, we asked them to provide a station title and description that would be made available to the public as well as an overview of their proposed activities and intended goals in hosting their station. A critical aspect of research outreach events for the public is ensuring that materials are accessible to all ages and education levels. Many zoos have designated education staff, and we relied on Zoo Atlanta’s education team to determine if station titles and descriptions were sufficiently accessible and engaging. For titles and descriptions deemed to contain too much jargon or high-level principles, we proposed edits that we believed would maintain the integrity of the intended goals of the station and passed these back to the research labs for approval. This iterative process could be performed several times, but it was imperative to have both the research labs and the zoo’s education team approve the materials. Most labs also proposed interactive activities, and these likewise needed to be approved by the zoo to ensure public safety.

**Fig. 3:**
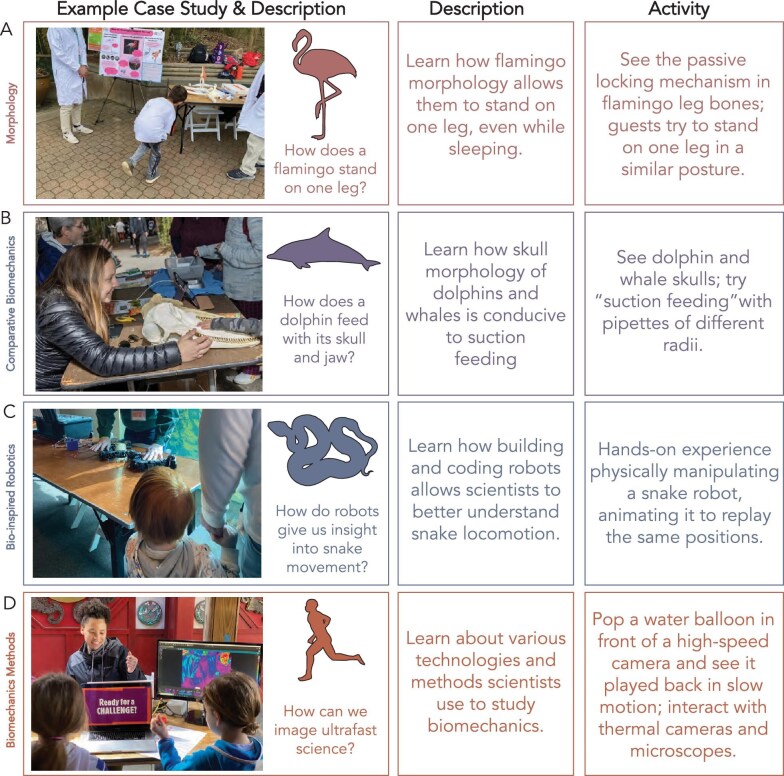
Four themes of demonstrations of academic booths at the event. Examples stations with corresponding descriptions and activities representing four key themes, including Morphology, Comparative Biomechanics, Bio-inspired robotics, and Biomechanics Methods.

The biomechanics stations at Zoo Biomechanics Day are not restricted to a specific theme, much like the field of biomechanics, but instead represent a spectrum of research investigating different structures, functions, and mechanisms of flora and fauna using diverse interdisciplinary techniques ranging from neuromechanics of jumping and flight to traversing rough and complex terrains with a bio-inspired robot. The goal of the breadth of these stations was to highlight not just the biodiversity of nature but also the diversity of biomechanics as a research field. Overall, the stations featured in this event had four primary themes, each of which we will highlight with a case study (Fig. 3):

1. Morphology—standing like a flamingo, inspired by Chang and Ting (2017).
2. Comparative biomechanics—marine-mammal biomechanics, inspired by  Page and Cooper (2017).
3. Bio-inspired robotics—snake-like robotic movement for complex terrain inspired by  Marvi et al. (2013).
4. Biomechanics methods—ultra-fast locomotion and high speed cameras inspired by Ortega-Jimenez et al. (2025).

**Morphology:** The form–function paradigm of biomechanics often revolves around fundamental morphology. One such example of a morphology-based research collaboration with Zoo Atlanta is studying how flamingos are able to stand on one leg, even when sleeping. Georgia Tech researchers in the Chang Lab created a biomechanics station highlighting some of their results (Chang and Ting 2017). Lab members brought 3D-printed flamingo femur and tibiotarsus bones and used a poster with additional visuals to show guests how the passive locking mechanism works in the birds—and why a similar stance in humans would be much more energetically costly. To solidify the latter point, the station volunteers also kept a running contest throughout the event to see how long guests could stand on one leg while crouching, with some kids checking on their standings later in the day. By hosting this station in front of the flamingo habitat at the zoo, guests had ability to observe the animals live while learning about their underlying morphology of leg bones.

**Comparative biomechanics:** The Blob Lab at Clemson University conducts research that compares animal feeding and locomotor performance across species, with a focus on how these systems work in aquatic habitats. Station volunteers brought casts of sample dolphin and whale skulls to discuss how the morphology is conducive to suction feeding in water. Casts were preferred over skulls because attendees, especially kids, could actually touch and interact with them. The idea of morphology-driven suction feeding was further emphasized with an interactive suction-feeding demonstration where guests used pipettes with openings of different radii to see how the negative pressure in a dolphin or whale mouth can suck in prey without having to make contact. The researchers also explained how this strategy is not viable in air because of its low density and viscosity.

**Bio-inspired robotics:** Another facet of biomechanics research involves using robotic models—isolating certain behaviors and other variables to elucidate locomotor principles in their organism counterparts. One such lab showcasing this technique was the Astley Lab from the University of Akron. Directly in front of a selection of reptile and amphibian habitats at the zoo, this station featured snake-like robots that guests could program themselves. Guests could manually pose a robot, record the poses, then observe the robot cyclically moving through the recorded poses like in stop-motion animation. While getting this hands-on experience, guests also got to hear from the researchers about bio-inspired robots and ongoing snake locomotion research (Marvi et al. 2013). Once again, having this station in close proximity to related habitats allowed guests to see both ongoing research methods in robotic versions of an animal as well as the live version, perfectly marrying the guest experience with biomechanics research outreach.

**Biomechanics methods:** A critical first step in biomechanics research involves the selection and usage of appropriate experimental tools and technology. The Bhamla Lab from Georgia Tech created a biomechanics station featuring some of these tools, providing zoo guests the opportunity to personally collect new data. Researchers set up high-speed cameras, allowing guests to pop a water balloon and immediately watch the burst played back. Guests also interacted with a real-time heat camera, comparing individual and anatomical differences in thermal intensities of their hands and faces with each other. Ultimately, this methods station enabled guests to personally experience data collection techniques, connecting them to a fundamental process of biomechanics research.

### Execution and outcomes of the event

Each year, Zoo Atlanta budgets approximately $300 that is used to purchase items such as extension cords, stamps for the maps, and snacks for the presenters, and to print ID-tags for the presenters and maps for the guests. Many purchased items are reusable across multiple years.

The first year the event ran (2023), the zoo welcomed 7642 guests, which is 65% greater than average visitation on comparable days and weather in the 3 years ($n=10$) prior to launching the event. Of these 7642 guests, 1168 were specifically registered with Educator Appreciation Day, leaving a 3011 guest increase associated with our ZBD event. In 2024, the second iteration of ZBD occurred with 11 labs from 5 universities and a representative from a biomechanics tech company. The weather for this event was rainy and the total number of guests was 3436. Although this is lower than the previous year’s number of guests, this is still 60% higher than the average rainy day attendance at the zoo, and the weather was predicted to be thunderstorms up until that morning. The third event year (2025), we had 9 labs from 5 universities and a representative booth from a student organization. A total of 6828 guests came to the zoo, highlighting a 48% greater than the average visitation day. The fourth event year (2026), we had 8 labs from 3 universities and two booths from student organizations with an attendance of 8844, making the average guest increase across all 4 years regardless of weather 65.8% $\pm$ 15.8%.

It was not possible to record exactly how many guests participated in the presentation booths due to the high number of guests and the added staff time that would be required to monitor the number of guests engaging with each station. However, a number of attending presenters qualitatively reported a continuous stream of guests at their booths. One PI approximated their booth generated around 240 interactions during one event, with the disclaimer that this number is likely an underestimate as groups were often larger than one individual. Even as an underestimate, this approximation would yield at least 2500 total interactions across 10 booths during a single event.

The reach and influence of a STEAM outreach-education program can be enormous when a large academic research university collaborates with an accredited zoo. In each of the 4 years of running ZBD, roughly 30 volunteers from university labs presented their science, and most of the volunteers were graduate and undergraduate students. In total, 10 principal investigators (PIs) and two student organizations have come from 5 different universities, resulting in 17 unique stations across the 4-year period. Attendance totaled 26,750 guests across all 4 years of the event, with a significant difference in zoo attendance for this outreach day compared to other days.

## Discussion

In this paper, we highlight an outreach event pathway that allows university-zoological partnerships to reach thousands of people representing a broad demographic diversity with minimum individual work and minimal overall cost, while maximizing community engagement and impact. Our event continues to grow annually, and the primary goal is to keep expanding early access to interdisciplinary scientific fields such as biomechanics and bio-inspired design to show the research impact zoo-academic collaborations can have. Hosting these outreach events is a win–win–win scenario for the parties involved.

### Making your event annual

While creating the first event required several meetings between Zoo Atlanta and Georgia Tech organizers to plan how to run the event, we were able to easily adapt existing materials for future years. When planning the first event, we created and maintained shared drives for all documents both on the planning side (records of which labs participated, their station titles and descriptions, contact information, etc.) and the public side (the zoo map, station title posters, and all other printed materials). When we started planning the second event, we simply duplicated the files into a new folder and updated them with the new information. Because both Zoo Atlanta and science volunteers felt pleased with the the set-up and flow of the first event, fewer meetings were required to organize the second one. We hosted an organizer meeting 6 months prior to the second event to confirm interest and the date, 4 months prior to outline the tasks to be accomplished, and 2 months to confirm which labs were participating and station placement. Final meetings were held 1 month, 2 weeks, and 1 week prior to confirm final logistics (how many extension cords, tables, and chairs at each station, etc.) and ensure all science and Zoo Atlanta volunteers had the necessary information for the event day.

In addition to the logistics of planning, we also made sure to advertise the success of the first event to accumulate greater interest for future events. Zoo Atlanta appreciated the scientists showing respect for their staff, animals, and space, and asked if academic participants would be willing to return the next year as they were leaving the first event. We obtained attendance numbers from Zoo Atlanta’s admissions and were able to share these with lab volunteers for inclusion in their outreach reports. We also created an online drive shared between lab volunteers to upload pictures taken by themselves and Georgia Tech organizers. Between their experiences and additional scientific connections, we were able to recruit even more labs for the second event.

### A win for all parties

**Fig. 4:**
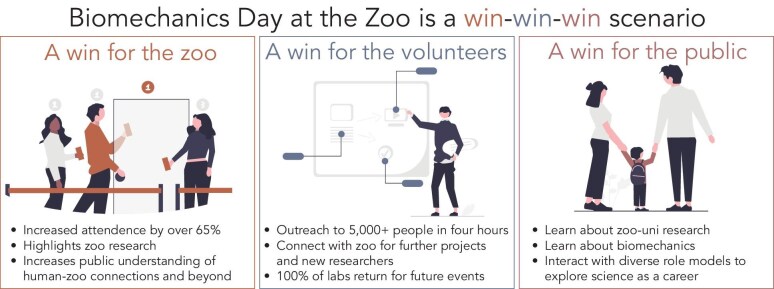
Outcomes of the event highlighting across collaborative benefits. Highlighting the win–win–win scenario of hosting a large-scale collaborative zoo-academic outreach event.

**A win for the zoo:** In all four instances of hosting this event, the zoo had increased numbers from similar types of scenarios (e.g., a rainy Saturday). The increase in numbers allows additional people to support the zoo. Several of the research projects highlighted are now being researched at or around the zoo with personnel, and hosting these events can help with the PR for these specific projects. In our case, we were able to highlight the long-standing Zoo Atlanta–Georgia Tech collaborations. Furthermore, Zoo Atlanta’s mission statement is: “We save wildlife and their habitats through conservation, research, education and engaging experiences. Our efforts connect people to animals and inspire conservation action.” This event allows the zoo to achieve their mission by creating greater connections to science and highlighting the importance of preserving wildlife and wild places. Zoo Atlanta is able to highlight the importance of science and collaboration while providing a venue for guests to engage with scientists they would not normally meet or see (Fig. 4).

**A win for the volunteers:** The volunteers at our event were lab personnel from research institutions, including faculty, postdocs, research scientists, graduate students, and undergraduate researchers. Interacting with so many zoo guests allowed volunteers to disseminate their research in a fun and engaging way, practice and hone their science-communication skills for broad audiences, and help improve public perception of science by making it more accessible, all while only requiring a relatively low amount of work from each individual. The time commitment for each lab took a maximum of 2–3 h of preparation and setup, with the event itself lasting only a few hours. Many volunteers also reuse station designs and materials each year, further reducing the time commitment. Outreach events are also one of the best ways to start a research partnership with a zoo (Schulz et al. 2022). In some of the cases, lab-group volunteers were able to interface with keepers and zoo research personnel before and after the event to discuss future research ideas and plans that stemmed from the outreach day. Specific to Biomechanics Day at the Zoo, there have been several collaborative partnerships formed between Zoo Atlanta and Georgia Tech. Beyond the event, many of the Ph.D. students and postdocs that volunteered have moved on to scientific opportunities outside of Atlanta and have started to interact with their local zoos. At this time, we are aware of several new events or research collaborations inspired by the first event in Atlanta, including some in Tennessee, North Carolina, California, and Michigan.

Outreach also falls within “Broader Impacts” for resume building and grant reporting, helping all levels of volunteers highlight their community involvement. In summary, volunteers had to commit fairly minimal time for thousands of impacts, allowing them to develop and practice science-communication skills and potentially blossoming into future research collaborations and opportunities (Fig. 4).

**A win for the participants:** The biggest win for this outreach event is for those who attend and participate (Fig. 4). To our knowledge, this is one of the largest single-event biomechanics or bioinspiration event ever run. In 4 years, we have introduced 26,750 individuals to the field of animal biomechanics and how physicists, engineers, and biologists engage in the study of the complexities of evolution and biological function. One of the biggest connections we worked to leverage for participants is the connection between these types of research and the important biodiversity conservation work happening at the zoo facility. One of the biggest challenges in biomechanics and bio-inspired design is that students are not exposed to these fields of interest until very late in their education. By hosting events like this at zoological facilities, these fields can start to grow, with some of our stations accessible to elementary school kids.

**A win for longevity:** While the first year required making big-picture decisions about the event’s objectives, timing, and individual biomechanics stations, the time requirement for subsequent years was fairly minimal. We partnered with current scientific outreach festivals in the area (i.e., Atlanta Science Festival, https://atlantasciencefestival.org/), which only required a brief event proposal that is reusable each year. Because this event was hosted at a zoo, all logistics regarding food accommodations, ticketing, etc., are handled as part of the zoo’s normal operating procedure. Biomechanics stations are meant to showcase ongoing research, and many academic volunteers simply brought in current experimental materials, laptops to show pictures and videos, or existing posters. For labs that choose to create specific materials for this event, many of those materials are reusable (and are actively reused) in subsequent years. By saving and utilizing existing templates for spreadsheets, forms, and emails, event coordinators have streamlined the planning process. Finally, the Atlanta Science Festival did much of the marketing and communication through their website and various media partnerships. It’s also worth noting that other cities have similar events to the Atlanta Science Festival, such as the San Diego Festival of Science and Engineering and the World Science Festival in New York City, and one of the authors is currently looking to expand ZBD to Southern California.

### Lessons learned

After 4 years of running ZBD, we have identified several key features others should consider when planning their own major zoo-academic outreach event. One critical step is to be organized upfront with a shared drive that both the zoo and academic event coordinators have access to. When deciding station locations and creating event documents, it is critical to work with the zoo’s education team. They know the zoo layout, which habitats have access to electrical outlets, how to create effective public education materials, etc. We also recommend identifying a point person or two (from academia) to communicate with all academic volunteers to avoid overwhelming the zoo with redundant questions. Once these individuals are identified, it is critical to assign specific tasks and deadlines to members of the organizational team to minimize the number of meetings necessary and make the meetings as straightforward as possible.

## Conclusion

Outreach events planned with organizations such as zoos, aquaria, museums, and botanical gardens can host thousands of people in a short amount of time. These events expose the public to the new interdisciplinary fields stemming from these incredible science collaborators. Fields such as biomechanics, functional morphology, and bio-inspiration are rooted in collaborations and highlighting these collaborations in an outreach event can be a no-lose scenario, helping the volunteers and organization develop new research topics while displaying ongoing work to thousands. We encourage researchers around the globe to develop collaborative events with their local biological collaborators and organizations to help break the barriers-of-entry to these complex fields and help educate and inspire the next generation of outstanding biological interdisciplinary researchers and biodiversity advocates.

## Supplementary Material

obag022_Supplemental_Files
