# Supplementary figures and images for: Bridging Science Across Species: A Biomechanics Outreach Event at the Zoo
Source: Integr Org Biol. 2026 May 19;8(1):obag022. doi: 10.1093/iob/obag022 (PMC13270971; doi:10.1093/iob/obag022)

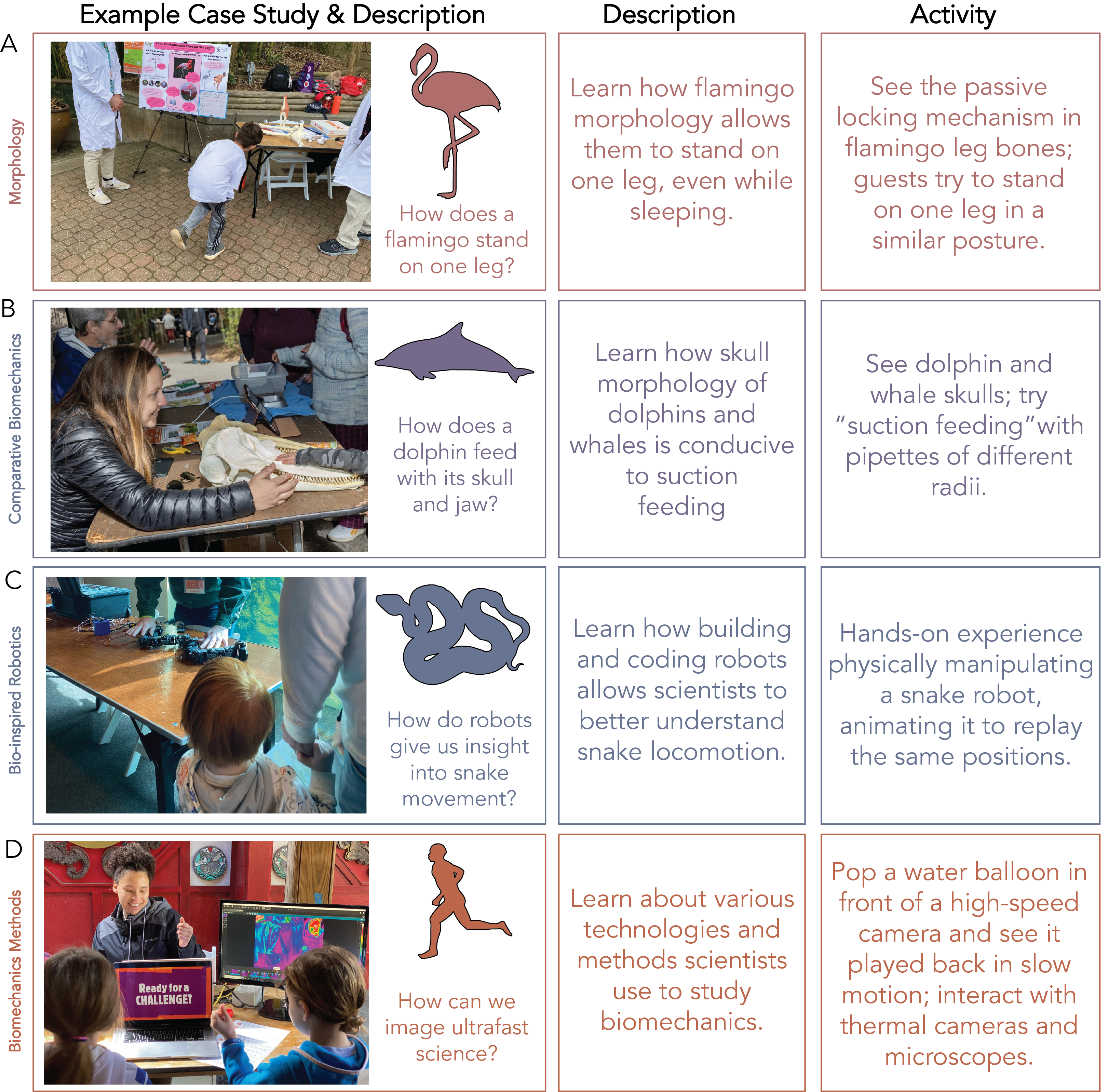

Supplement: obag022_Supplemental_Files [file obag022_supplemental_files.zip › DemoFigure3.png]

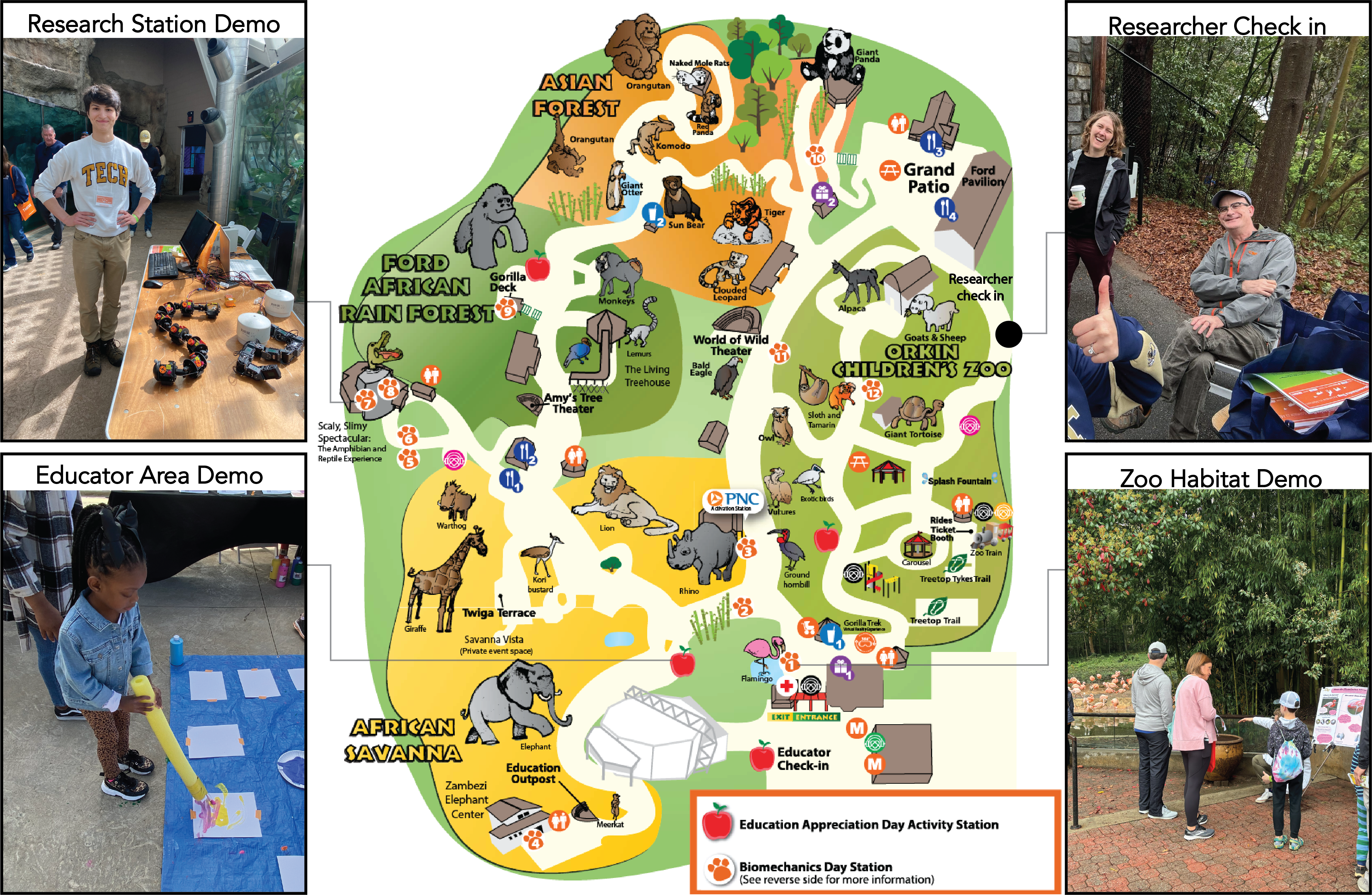

Supplement: obag022_Supplemental_Files [file obag022_supplemental_files.zip › ZBD_Figure1.png]

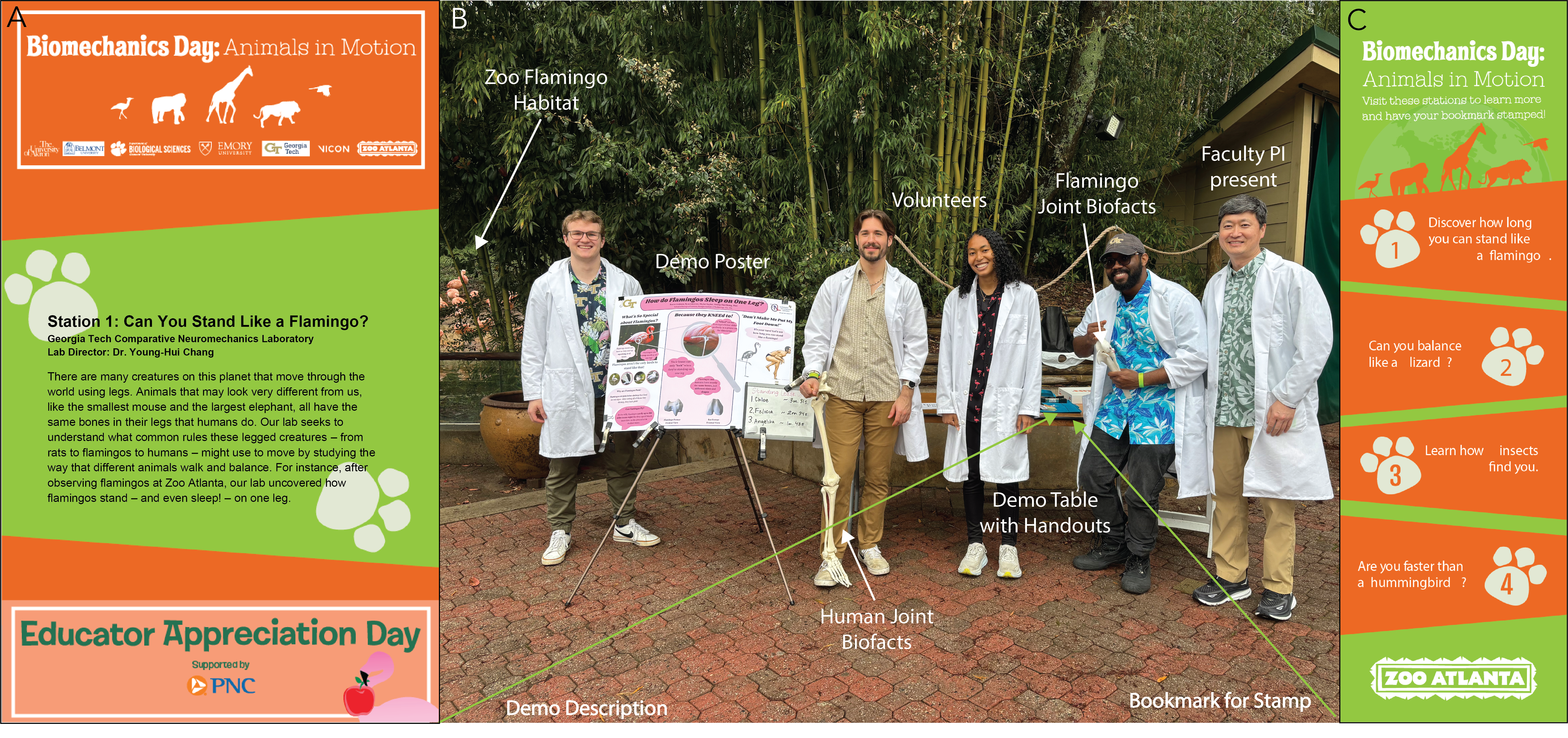

Supplement: obag022_Supplemental_Files [file obag022_supplemental_files.zip › ZBD_Figure2.png]

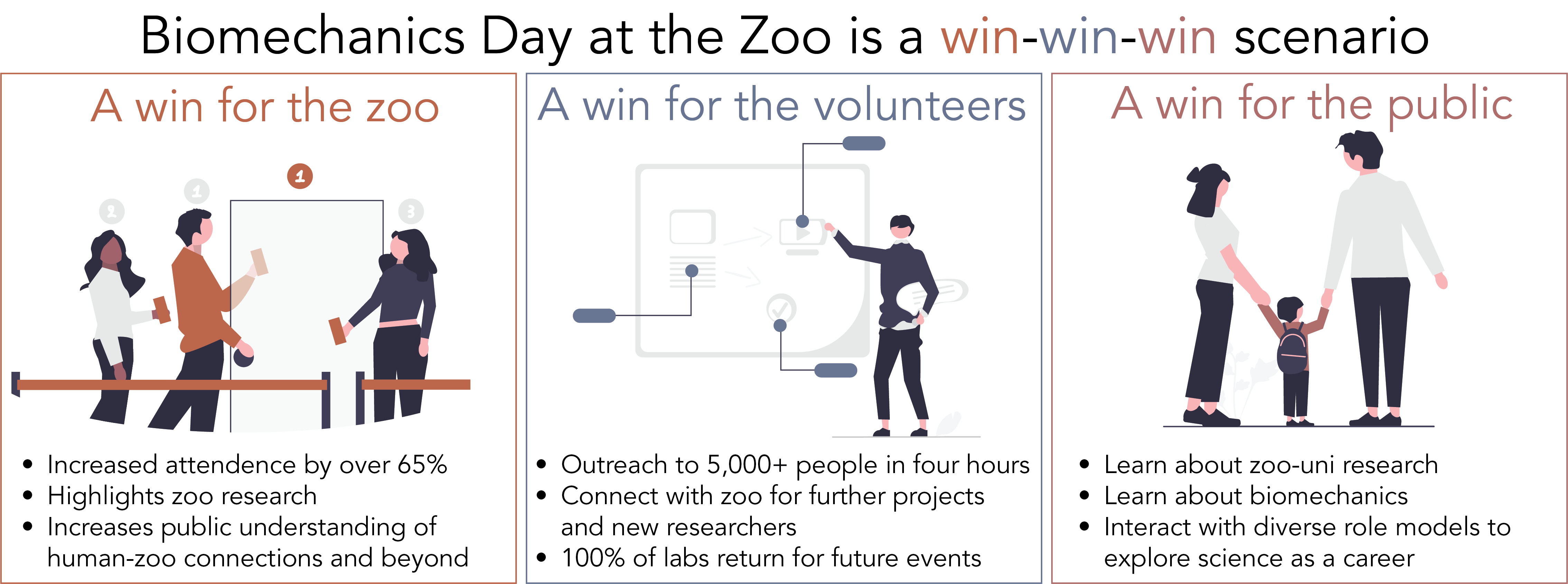

Supplement: obag022_Supplemental_Files [file obag022_supplemental_files.zip › ZBD_Figure4v2.png]
